# Synthesis, Crystal Structure and Interaction With DNA of N,N′-(Butane-1,4-Diyl)Bis(Guanidinium) Tetrachloroplatinate (II)

**DOI:** 10.1155/MBD.1998.279

**Published:** 1998

**Authors:** Christian Bailly, Bernard Viossat, Xavier Labouze, Georges Morgant, Carmella Saturnino, Jean Charles Lancelot, Max Robba, Nguyen Huy Dung

**Affiliations:** 1 Laboratoire de Pharmacologie Antitumorale du Centre Oscar Lambret and INSERM U-124 Place de Verdun Lille France; 2 Laboratoire de Chimie Générale Faculté Mixte de Médecine et de Pharmacie Poitiers France; 3 Laboratoire de Cristallochimie Bioinorganique Faculté de Pharmacie Paris XI Châtenay-Malabry Paris France; 4 Laboratoire de Biochimie Hôspital Armand Trousseau, AP-HP Paris France; 5 Centre d′Etude et de Recherche surle Médicament de Normandie UFR des Sciences Pharmaceutiques Caen France

## Abstract

The design, synthesis, crystal structure and interaction with DNA of the N,N′-(butane-1,4-diyl)bis(guanidinium) tetrachloroplatinate(ll) are described. Crystal data: a = 8.152(1), b = 8.889(4), c = 10.700(3) Å , α = 81.59(3), β = 87.99(5), γ = 78.48(6)°, V = 752(1) Å^3^, Z = 2 , space group P-1. The structure was refined to R = 0.039 and Rw = 0.046 from 1853 reflections (I > 3σ(I)). This compound, named PtC_4_Gua, does not exhibit a center of symmetry and the center linker chain C(2) - C(3) - C(4) - C(5) is in *gauche* conformation. The cation is bisprotonated with the H^+^ attached to the imine group of each terminal guanidinium function. The presence of the platinum moiety reinforces the binding of the butane(bis)guanidinium structure with double stranded DNA as judged from thermal denaturation studies and DNA unwinding experiments.


### SYNTHESIS, CRYSTAL STRUCTURE AND INTERACTION WITH DNA OF N,N'-(BUTANE-1,4-DIYL)BIS(GUANIDINIUM) TETRACHLOROPLATINATE (II)

Christian Bailly*[1], Bernard Viossat[2], Xavier Labouze[3], Georges Morgant[3,4], Carmella Saturnino[5], Jean Charles Lancelot[5], Max Robba[5] and Nguyen Huy Dung[3]

[1] Laboratoire de Pharmacologie Antitumorale du Centre Oscar Lambret and INSERM U-124, Place de Verdun, Lille, [2] Laboratoire de Chimie Générale, Faculté Mixte de Médecine et de Pharmacie, Poitiers, [3] Laboratoire de Cristallochimie Bioinorganique, Faculté de Pharmacie Paris XI, Châtenay-Malabry, [4] Laboratoire de Biochimie, Hôpital Armand Trousseau, AP-HP, Paris, and [5] Centre d'Etude et de Recherche sur le Médicament de Normandie, UFR des Sciences Pharmaceutiques, Caen, France

* Correspondence and reprint requests : E-mail: bailly@lille.inserm.fr FAX: (+33) 3 20 16 92 29

This paper is dedicated to the memory of Dr Daniel Perrine (deceased on May 25$^{th}$ 1997) who initiated our collaboration.

**Abstract** The design, synthesis, crystal structure and interaction with DNA of the N,N'-(butane-1,4-diyl)bis(guanidinium) tetrachloroplatinate(II) are described. Crystal data : a = 8.152(1), b = 8.889(4), c = 10.700(3) Å , $\alpha$ = 81.59(3), $\beta$ = 87.99(5), $\gamma$ = 78.48(6)°, V = 752(1) Å$^3$ , Z = 2 , space group P-1. The structure was refined to R = 0.039 and Rw = 0.046 from 1853 reflections ($I > 3\sigma(I)$). This compound, named $PtC_4Gua$, does not exhibit a center of symmetry and the center linker chain C(2) - C(3) - C(4) - C(5) is in *gauche* conformation. The cation is bisprotonated with the $H^+$ attached to the imine group of each terminal guanidinium function. The presence of the platinum moiety reinforces the binding of the butane(bis)guanidinium structure with double stranded DNA as judged from thermal denaturation studies and DNA unwinding experiments.

### Introduction

Platinum complexes, like *cis*-diamminedichloroplatinum (II) (cisplatin) and carboplatin, are effective antitumor agents used for the treatment of genitourinary and head and neck cancers [1]. Very recently, oxaliplatin (1*R*,2*R*-diaminocyclohexanedicarboxylatoplatinum(II)) has been recommended in metastatic colon rectal cancer treatment with neither nephrotoxicity nor cross-resistance to cisplatin [2]. The biological effects are attributed to the formation of adducts with DNA. Cisplatin-induced bifunctional intrastrand cross-links between neighbouring purine base residues induce marked conformational kinks in DNA. These lesions are considered relatively difficult to repair [3].

The severe side effects of cisplatin and the development of resistance have encouraged the design of alternative platinum compounds with a broader spectrum of activity, in particular against cisplatin-resistant cell lines. Over the last ten years, a large number of drugs containing the basic cis-$[PtX_2(amine)_2]$ motif were synthesized [3]. Dimers formed by linking the monofunctional platinum species $[Pt(dien)Cl]^+$ by a tetramethylene linker exhibit a greatly enhanced ability for interstrand cross-linking compared to cisplatin itself [4]. The bis(platinum) complex shown in Figure 1 represents a promising antitumor agent with a pronounced cytotoxic effect toward cell lines resistant to cisplatin [5]. Recently, a novel bifunctional triplatinum(II) complex, BBR 3464, was shown to exhibit comparable or superior efficacity to cisplatin and is now entering phase I clinical trials [6]. Its structure can be described as two *trans* $[PtCl(NH_3)_2]$ units linked by a central $NH_2(CH_2)_6NH_2$ - *trans* - $Pt(NH_3)_2$ - $NH_2(CH_2)_6NH_2$ diamine chain.

Several non-classical platinum complexes that do not contain the aforementioned basic motif have also been developed. Among them, the compound [rhodamine-123] $PtCl_4$ was the first tetrachloroplatinate (II) endowed with potent anticancer activity [7]. In addition, an $(ethidium)_2$ $PtCl_4$ conjugate showed reduced toxicity to mice compared to ethidium but equal trypanocidal activity [8]. These considerations prompted us to synthesize the bis(guanidinium) tetrachloroplatinate(II) salt, hereafter named $PtC_4Gua$, which incorporates a (butane-1,4-diyl)bis(guanidinium) cation and $PtCl_4^{2-}$ as counteranion.

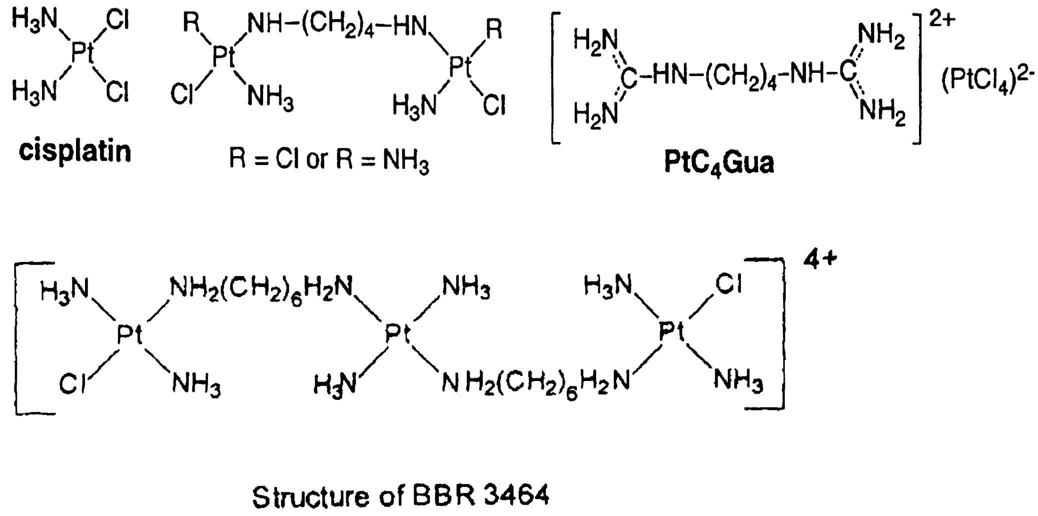

Figure 1: Structure of some platinum(II) compounds.

### 2 Results and discussion

2.1 Description of the N,N'-(butane-1,4-diyl)bis(guanidinium) tetrachloroplatinate (II) structure. Distances and angles are reported in Table 1. In the N,N'-(butane-1,4-diyl)bis(guanidinium) tetrachloroplatinate (II) $[C_6H_{18}N_6]^{2+}[PtCl_4]^{2-}$ (fig. 2), the asymmetric unit consists of one square planar $[PtCl_4]^{2-}$ anion and one N,N'-(butane-1,4-diyl)bis(guanidinium) cation.

Table 1: Interatomic distances (Å) and angles (°). s.d's in parentheses refer to the last significant digit.

| a) distances | | | b) angles | | | |
|---|---|---|---|---|---|---|
| Pt(1) | -Cl(1) | 2.308(2) | Cl(1) | -Pt(1) | -Cl(2) | 89.8(1) |
| Pt(1) | -Cl(2) | 2.301(3) | Cl(1) | -Pt(1) | -Cl(3) | 177.71(9) |
| Pt(1) | -Cl(3) | 2.303(2) | Cl(2) | -Pt(1) | -Cl(3) | 90.5(1) |
| Pt(1) | -Cl(4) | 2.302(3) | Cl(1) | -Pt(1) | -Cl(4) | 90.9(1) |
| N(12) | -C(11) | 1.34(1) | Cl(2) | -Pt(1) | -Cl(4) | 177.7(1) |
| N(13) | -C(11) | 1.31(1) | Cl(3) | -Pt(1) | -Cl(4) | 89.0(1) |
| N(14) | -C(1) | 1.50(1) | C(1) | -N(14) | -C(11) | 125.0(9) |
| N(14) | -C(11) | 1.31(1) | C(4) | -N(24) | -C(21) | 122.3(10) |
| N(22) | -C(21) | 1.33(1) | N(14) | -C(1) | -C(2) | 111.3(9) |
| N(23) | -C(21) | 1.29(1) | C(1) | -C(2) | -C(3) | 113.0(9) |
| N(24) | -C(4) | 1.49(1) | C(2) | -C(3) | -C(4) | 110.8(10) |
| N(24) | -C(21) | 1.31(1) | N(24) | -C(4) | -C(3) | 109.4(10) |
| C(1) | -C(2) | 1.54(2) | N(12) | -C(11) | -N(13) | 118.4(10) |
| C(2) | -C(3) | 1.53(1) | N(12) | -C(11) | -N(14) | 120.3(10) |
| C(3) | -C(4) | 1.48(2) | N(13) | -C(11) | -N(14) | 121.3(10) |
| | | | N(22) | -C(21) | -N(23) | 118.6(10) |
| | | | N(22) | -C(21) | -N(24) | 118.1(10) |
| | | | N(23) | -C(21) | -N(24) | 123.2(10) |

This cation does not exhibit an inversion center half away along the C(2)-C(3) bond and the central linker chain C(1) - C(2) - C(3) - C(4) exhibits a *gauche* conformation, as shown by the torsion angle value of -64.14°. In contrast, in the crystal structure of S,S'-(1,8-octanediyl)bis(thiouronium)-tetrachloroplatinate(II), the cation exhibits a centre of symmetry and packs in a mixed *trans*(*t*) and *gauche*(*g*) conformation, with a *tgtttttgt* sequence [9]. However, in the S,S'-(1,4-

butanediyl)bis(thiouronium) tetrachloroplatinate (II),the cation exhibits an extended *trans* configuration [10]. Moreover, in the title compound, the cation is bisprotonated with the $H^+$ attached to the imine group of each terminal guanidinium function and with the three equivalent C-N bonds in the range from 1.31(1) to 1.34(1) Å. The two guanidinium moieties are planar with the C(11) and C(21) out-of-plane displacements of -0.01 and 0.02 Å respectively and the dihedral angle is 20.4° between them. In the $[PtCl_4]^{2-}$ counteranion, the platinum atom exhibits a quasi-ideal square planar coordination, the distances being in the range 2.301(3)-2.308(2) Å and the angles 89.01-90.9(1)°. There is an extensive hydrogen-bonding network involving the four chloride atoms of each tetrachloroplatinate(II) anion and hydrogen atoms in the different guanidinium moieties (Table 2).

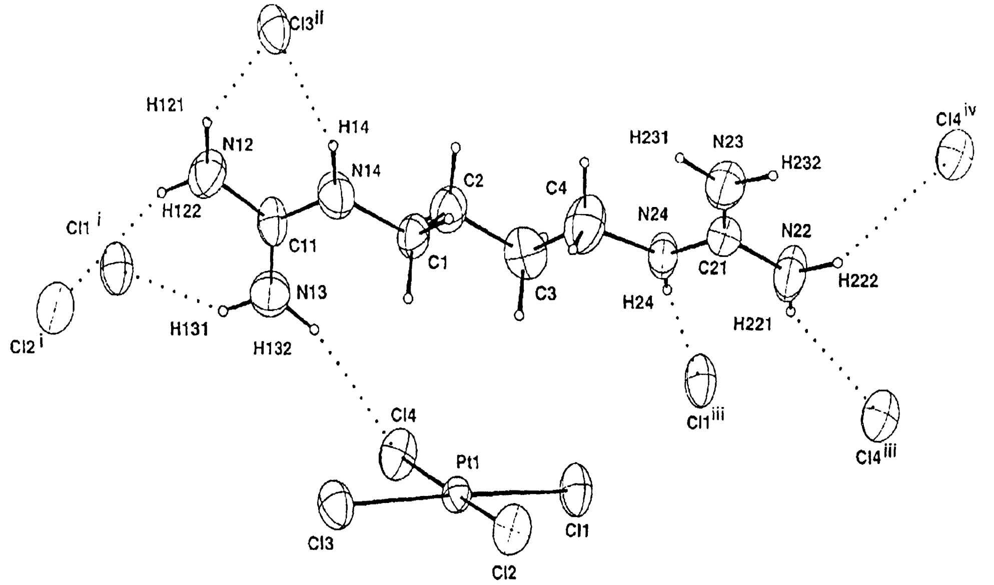

Figure 2 : Perspective view of $PtC_4Gua$ generated by CAMERON using 50% probability ellipsoids for the non-hydrogen atoms. The crystallographic labelling scheme is shown. The hydrogen bonds are shown by dotted lines. Symmetry code i: x, 1+y, z ; ii : -x, 1-y, 1-z ; iii : 1-x, -1-y, -z ; iv : 1+x, -1+y, z.

Table 2 : Hydrogen bonds (Å). E.s.d's in parentheses refer to the last significant digit.

| | | | Distance (Å) | Angle (°) |
|---|---|---|---|---|
| N(12) | -H(122) .... | $Cl(2)^{i}$ | 3.38(2) | 135 |
| N(13) | -H(131) .... | $Cl(1)^{i}$ | 3.38(2) | 146 |
| N(14) | -H(14) ...... | $Cl(3)^{ii}$ | 3.36(1) | 158 |
| N(22) | -H(221) .... | $Cl(4)^{iii}$ | 3.31(2) | 140 |
| N(22) | -H(222) .... | $Cl(4)^{iv}$ | 3.38(1) | 138 |
| N(24) | -H(24) ..... | $Cl(1)^{iii}$ | 3.36(1) | 169 |

Symmetry Code : i : x, 1+y, z ; ii : -x, 1-y, 1-z ; iii : 1-x, -1-y, -z ; iv : 1+x, -1+y, z

2.2 DNA binding

2.2.1 DNA thermal denaturation

The ability of the platinated compound $PtC_4Gua$ and its non-platinated analogue $C_4Gua$ to alter the thermal denaturation profile of double stranded DNA was used as a first indication of their propensity to bind to DNA.

The $\Delta Tm$ values are collated in Table 3. A much larger increase in the Tm of nucleic acids is observed with the platinated compound than with the Pt-free bisguanidine.

The stabilization of the DNA double helix by binding of the drug increases with increasing molar ratio of drug to DNA-phosphate (D/P). We can conclude that the bisguanidinium chain promotes the interaction with DNA and that the $[PtCl_4]^{2-}$ anion contributes to reinforce the interaction with DNA.

Table 3 . Variation in melting temperature

| | | $C_4$Gua | | $PtC_4$Gua | |
|---|---|---|---|---|---|
| | conc (μM) | 10 | 20 | 10 | 20 |
| (ΔTm in °C) | poly $(dA\text{-}dT)_2$ | 3.6 | 9.0 | 19.2 | 25.2 |
| (ΔTm in °C) | calf thymus DNA | 1.6 | 10.6 | 9.7 | 13.6 |

2.2.2 DNA double helix unwinding

We determined the capacity of the drug to unwind the double helix. The antitumor drug cisplatin readily unwinds supercoiled DNA by about 13° corresponding to covalent crosslinking into the double helix [11]. The local duplex unwinding is believed to be a major determinant in the recognition of DNA damages by repair enzymes and therefore it may be essential to the biological activity of the drug. Electrophoresis in native agarose gel was used to determine the unwinding induced in pUC19 plasmid by $PtC_4$Gua by monitoring the degree of supercoiling. This sensitive method has been used previously to quantify the unwinding produced by cisplatin and a variety of platinum complexes [12].

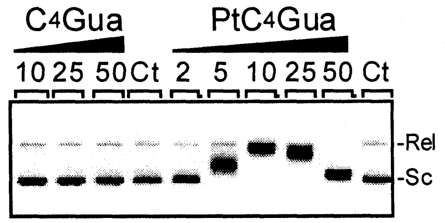

Figure 3 : Unwinding of supercoiled pUC19 plasmid DNA by PtC4Gua. Sc, supercoiled DNA ; Rel, relaxed DNA.

As shown in Figure 3, the number of supercoils in the plasmid is reduced upon addition of $PtC_4$Gua whereas there is absolutely no effect with the analogue $C_4$Gua lacking the platinum groups as well as with $K_2(PtCl_4)$. Supercoiled DNA becomes progressively relaxed as the double helix is unwound by $PtC_4$Gua. When the supercoiled DNA comigrates with the nickel relaxed band, the DNA has been fully relaxed. This coalescence point is used to determine the amount of drug necessary for complete removal of all supercoils from the DNA and then to calculate the unwinding angle. Under these conditions of the present experiments, we calculated an unwinding angle of 8 (1° for $PtC_4$Gua which is lower than the angle determined for cisplatin (12 (1°)) but, however, it falls in the range of unwinding angles commonly determined with platinated compounds [12]. Together with the Tm measurements, these experiments leave no room for doubt that the platinum moiety of $PtC_4$Gua plays a significant role in the interaction with DNA. The design of small molecules containing a tetrachloroplatinate(II) center may represent a valuable approach to the conception of new classes drugs acting at the level of DNA.

3 Experimental

3.1. Synthesis

Reaction of 1,4-diaminobutane with 3,5-dimethylpyrazole-1-carboxamidine nitrate in ethanol under reflux afforded N,N'-(butane-1,4-diyl)bis guanidinium nitrate ($C_4$Gua) in a 71% yield.

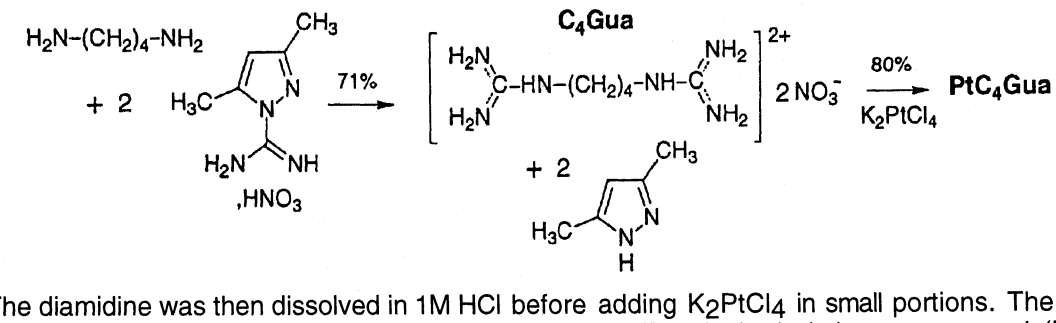

The diamidine was then dissolved in 1M HCl before adding $K_2PtCl_4$ in small portions. The solution was heated for 2 hours at 60°C and then cooled down . The desired platinum compound ($PtC_4$Gua) was obtained in a 80% yield by slow evaporation of the solution at room temperature.

3.2 Structure determination of $PtC_4Gua$ $[C_6H_{18}N_6]^{2+}[PtCl_4]^{2-}$

The refined cell constants and other relevant crystal data are presented in Table 4, together with details of the intensity measurements. The crystal was mounted, using glass fibers, on an ENRAF-NONIUS CAD4 diffractometer equipped with a graphite monochromator. The lattice parameters were refined using 25 reflections. The data were collected using the $\omega$-$2\theta$ scan technique and with Mo K$\alpha$ radiation ( $\lambda$ = 0.71073 Å ).

Table 4: Crystal data for the title compound.

| | |
|---|---|
| **Crystal Parameters** | |
| compound | $[C_6H_{18}N_6]^{2+}[PtCl_4]^{2-}$ |
| fw(g) | 511.15 |
| shape (colour) | parallelepiped (orange) |
| size, mm | 0.05, 0.08, 0.30 |
| crystal system | triclinic |
| space group | P -1 |
| a, Å | 8.152(1) |
| b, Å | 8.889(4) |
| c, Å | 10.700(3) |
| $\alpha$, ° | 81.59(3) |
| $\beta$, ° | 87.99(5) |
| $\gamma$, ° | 78.48(6) |
| V, $Å^3$ | 752(1) |
| Z | 2 |
| F(000) | 480.26 |
| $\rho$(calcd), g.$cm^{-3}$ | 2.26 |
| $\mu$ (MoK$\alpha$), $cm^{-1}$ | 101.3 |
| **Data collection** | |
| Diffractometer | Enraf-Nonius CAD4 |
| monochromator | graphite |
| radiation, Å | MoK$\alpha$ ($\lambda$ = 0.71073) |
| Scan mode | $\omega$ - $2\theta$ |
| temperature, K | 291 |
| $2\theta$ range, deg | $4.0 < 2\theta < 48$ |
| Absorption correction | None |
| no. of unique rflns | 2351 |
| reflections used | 1853($I > 3\sigma(I)$) |
| **Refinement** | |
| R / $R_W$ | 0.039 / 0.049 |
| Weighting Scheme | Chebyshev |
| Coefficient Ar | 3.30,-0.276,2.64 |
| GOF | 1.13 |
| $(\Delta/\sigma)_{max}$ | 0.03 |
| $\Delta\rho_{min}/\Delta\rho_{max}$ (e. $Å^{-3}$) | -1.41/1.61 |
| Number of parameters | 155 |

During the data collection, three intensity control reflections were monitored every two hours, showing no loss of intensity. The data were corrected for Lorentz and polarisation effects. The structure was solved by a combination of direct methods using SIR procedure [13] and heavy-atom techniques and refined by full-matrix least-squares method based on F, using CRYSTALS [14]. No absorption correction was applied as $\mu R$ was estimated equal 0.3, where R is half the minimum crystal dimension. Anisotropic displacement parameters were assigned to all non-H atoms. The hydrogen atoms were introduced in calculated idealized positions (d(C-H) = 0.96 Å) and their atomic coordinates were recalculated after each cycle.

They were given isotropic thermal parameters 20% higher than those of the carbon to which they are attached. Least-squares refinements were performed by minimizing the function $\Sigma w(|F_o|-|F_c|)^2$, where $F_o$ and $F_c$ are the observed and calculated structure factors. The weighting scheme used in the last refinement cycles was $w = w'[1-(\Delta F/6\sigma(F_o)^2]^2$ where $w' = 1/\Sigma_1^n A_r T_r(x)$ with 3 coefficients $A_r$ for the Chebyshev polynomial $A_r T_r(x)$ where x was $F_c/F_c(max)$ [15]. Models reached convergence with $R = \Sigma(||F_o|-|F_c||)/\Sigma(|F_o|)$ and $Rw = [\Sigma w(|F_o|-|F_c|)^2/\Sigma w(F_o)^2]^{1/2}$, having values listed in Table 3. Criteria for a satisfactory complete analysis were ratios of rms shift to standard deviation less than 0.1 and no significant features in final difference maps. Details of data collection and refinement are given in Table 4. Calculations were performed with a PC CRYSTALS package program. The drawings of the molecules were generated using CAMERON [16]. The atomic scattering factors were taken from International Tables for X-ray Crystallography [17].Fractional atomic coordinates and equivalent isotropic thermal parameters were shown in table 5.

Table 5 : Fractional atomic coordinates and equivalent isotropic thermal parameter U(eq). S. d's in parentheses refer to the last significant digit.
U(eq) is defined as the cube root of the product of the principal axes.

| Atom | x/a | y/b | z/c | U(eq) |
|---|---|---|---|---|
| Pt(1) | 0.23167(4) | -0.28103(4) | 0.27121(3) | 0.0291 |
| Cl(1) | 0.3462(4) | -0.4587(3) | 0.1393(2) | 0.0473 |
| Cl(2) | 0.2133(4) | -0.4814(3) | 0.4303(2) | 0.0472 |
| Cl(3) | 0.1071(3) | -0.1032(3) | 0.3993(2) | 0.0461 |
| Cl(4) | 0.2590(4) | -0.0786(3) | 0.1162(2) | 0.0491 |
| N(12) | 0.583(1) | 0.280(1) | 0.3871(9) | 0.0521 |
| N(13) | 0.428(1) | 0.165(1) | 0.272(1) | 0.0571 |
| N(14) | 0.656(1) | 0.018(1) | 0.3804(9) | 0.0489 |
| N(22) | 0.911(1) | -0.799(1) | 0.117(1) | 0.0567 |
| N(23) | 1.007(1) | -0.713(1) | 0.283(1) | 0.0534 |
| N(24) | 0.834(1) | -0.545(1) | 0.139(1) | 0.0461 |
| C(1) | 0.651(1) | -0.128(1) | 0.328(1) | 0.0429 |
| C(2) | 0.732(1) | -0.126(1) | 0.196(1) | 0.0473 |
| C(3) | 0.723(1) | -0.271(1) | 0.136(1) | 0.0527 |
| C(4) | 0.822(2) | -0.412(2) | 0.210(1) | 0.0627 |
| C(11) | 0.555(1) | 0.152(1) | 0.347(1) | 0.0422 |
| C(21) | 0.920(1) | -0.683(1) | 0.181(1) | 0.0441 |

3.3 DNA thermal denaturation

We measured the change of the absorbance at 260 nm as a function of the temperature for both calf thymus DNA (42% AT base pairs) and the synthetic polynucleotide poly(dA-dT)$_2$ in the absence and presence of the test drugs. The variation of the Tm of helix-to-coil transition of the two nucleic acids were determined in the presence of 10 and 20μM drug using 20 μM DNA.
Tm measurements were performed in BP buffer pH 7.1 (6 mM $Na_2HPO_4$, 2 mM $NaH_2PO_4$ ) using 20 μM DNA at 260 nm with a heating rate of 1°C/min. Each drug concentration was tested in duplicate. Tm for DNA alone : 42.5 and 58.4 °C for poly(dA-dT)$_2$ and calf thymus DNA, respectively.

3.4 Unwinding of supercoiled pUC19 plasmid DNA by PtC$_4$Gua.

The DNA (0.5 μg) was incubated with C$_4$Gua or PtC$_4$Gua at the indicated concentration (μM) for 3h at 37°C before loading the samples on a 1% agarose gel. Control lanes Ct refer to the plasmid DNA incubated without drug. After 2h electrophoresis, the gel was stained with ethidium bromide (1μg/ml) then destained in water prior to being photographed under UV light.

**References**

1. Abrams, M.J., Murrer, B.A. *Science*, **261**, 727 (1993). Reed, E., Dabholkar, M., Chaber, B.A. In *Cancer Chemotherapy and Biotherapy*, Chaber, B.A., Longo, D.L., Eds., Lippincott-Raven Pub., New York, 357 (1996).

2. Machouer, D., Diazrubio, E., Degramont, A., Schilf, A., Gastiaburu, J.J., Brienza, S., Itzhaki, M., Mertger, G., Ndaw, D., Vignoud, J., Abad, A., Francois, E., Gamelin, E., Marty, M., Sastre, J., Seitz, J.F., Chou, M., *Ann. Oncol.*, **7**, 95 (1996). Mathe, G., Kidani, Y., Segiguchi, M., Eriguchi, M., Fredj, G. *Biomed. Pharmacother.* , **43**, 237 (1989).
3. Comess, K.M., Lippard, S.J. In *Molecular Aspects of Anticancer-drug DNA Interactions*, Neidle, S., Waring, M.J., Eds., CRC Press: Boca Raton, 1993, chapter 5, Farrell, N. In *Advances in DNA Sequence-Specific Agents*, Vol. 3, Palumbo, M., Ed., JAI Press Inc. London, 179-199 (1998).
4. Farrell, N., de Almeida, S.G., Skov, K.A. *J. Am. Chem. Soc.* 110, 5018 (1988). Qu, Y., Farrell, N. *J. Am. Chem. Soc.* 113, 4851 (1991).
5. Roberts, J.D., Van Houten, B., Qu, Y., Farrell, N.P. *Nucleic Acids Res.* **17**, 9719 (1989). Farrell, N., Qu, Y., Feng, L., Van houten, B. *Biochemistry*, **29**, 9522 (1990).
6. Farrell, N.; Menta, E.; Valsecchi, M.; Di Domenico, R.; Da Re, G.; Manzotti, C.; Pezzoni, G.; Giuliani, F.C.; Spinelli, S. *J. Inorg. Biochem.* **67**, 173 (1997). Qu, Y.; Farrell, N.; Kasparkova, J.; Brabec, V. *J. Inorg. Biochem.* **67**, 174 (1997).
7. Abrams, M.J., Picker, D.H., Fackler, P.H., Lock, C.J.L. Haward-Lock, H.E., Faggiani, R., Teicher, B.A., Richmond, R.C. *Inorg. Chem.* **25**, 3980 (1986).
8. Farrell, N.P., Williamson, J., McKaren, J.J.M. *Biochem. Pharmacol.* **33**, 961 (1984).
9. Nguyen-Huy, D., Viossat, B., Lancelot, J.C. *Acta Cryst.* **C50**, 1434 (1994).
10. Nguyen-Huy, D., Rodier, N., Viossat, B., Lancelot, J.C. *Acta Cryst.* **C50**, 1574 (1994).
11. Cohen, G.L., Bauer, W.R., Barton, J.K., Lippard, S.J. *Science*, **203**, 1014 (1979); Bellon, S.F., Coleman, J.H., Lippard, S.J. *Biochemistry*, **30**, 8026 (1991.
12. Keck, M.V., Lippard, S.J. J. *Am. Chem. Soc.*, **114**, 3386 (1992).
13. Altomare, A., Cascarano, G., Giacovazzo G., Guagliardi A., Burla M. C., Polidori, G. and Camalli, M. SIR92 - a program for automatic solution of crystal structures by direct methods. *J. Appl. Cryst.* **7**, 435 (1994).

14 Watkin, D.J., Prout, C.K., Carruthers, J.R. and Betteridge, P.W. *CRYSTALS Issue 10*, Chemical Crystallography Laboratory, University of Oxford, Oxford, (1996).

15 Prince, E., *Mathematical Techniques in Crystallography*, Berlin, Springer-Verlag, (1982).

16 Watkin,D.J., Prout, C.K., Pearce, L.J, *CAMERON*, Chemical Crystallography Laboratory, University of Oxford, Oxford, (1996).

17 *International Tables for X-ray Crystallography*, Kynock Press, Birmingham, England, Vol IV, pp. 99 (1974).

**Received: July 6, 1998 - Accepted: July 9, 1998 -**
**Received in revised camera-ready format: September 9, 1998**

